# *TERT* expression is susceptible to BRAF and ETS-factor inhibition in *BRAF*^*V600E*^*/TERT* promoter double-mutated glioma

**DOI:** 10.1186/s40478-019-0775-6

**Published:** 2019-08-07

**Authors:** Lisa Gabler, Daniela Lötsch, Dominik Kirchhofer, Sushilla van Schoonhoven, Hannah M. Schmidt, Lisa Mayr, Christine Pirker, Katharina Neumayer, Carina Dinhof, Lucia Kastler, Amedeo A. Azizi, Christian Dorfer, Thomas Czech, Christine Haberler, Andreas Peyrl, Rajiv Kumar, Irene Slavc, Sabine Spiegl-Kreinecker, Johannes Gojo, Walter Berger

**Affiliations:** 1 Comprehensive Cancer Center-Central Nervous System Tumors Unit, Medical University of Vienna, Spitalgasse 23, BT86/E 01, 1090 Vienna, Austria; 2 Institute of Cancer Research, Department of Medicine I, Medical University of Vienna, Borschkegasse 8A, 1090 Vienna, Austria; 3 Department of Pediatrics and Adolescent Medicine, Medical University of Vienna, Waehringer Guertel 18-20, 1090 Vienna, Austria; 4 Department of Neurosurgery, Kepler University Hospital, Johannes Kepler University, Neuromed Campus, Wagner-Jauregg-Weg 15, 4020 Linz, Austria; 5 Department of Neurosurgery, Medical University of Vienna, Waehringer Guertel 18-20, 1090 Vienna, Austria; 6 Institute of Neurology, Medical University of Vienna, Waehringer Guertel 18-20, 1090 Vienna, Austria; 7 Division of Molecular Genetic Epidemiology, German Cancer Research Center, Im Neuenheimer Feld 580, 69120 Heidelberg, Germany

**Keywords:** *BRAF*, *TERT* promoter, Glioma, Brain tumor, ETS-factors, *ETS1*

## Abstract

The *BRAF* gene and the *TERT* promoter are among the most frequently altered genomic loci in low-grade (LGG) and high-grade-glioma (HGG), respectively. The coexistence of *BRAF* and *TERT* promoter aberrations characterizes a subset of aggressive glioma. Therefore, we investigated interactions between those alterations in malignant glioma. We analyzed co-occurrence of *BRAF*^*V600E*^ and *TERT* promoter mutations in our clinical data (*n* = 8) in addition to published datasets (*n* = 103) and established a *BRAF*^*V600E*^-positive glioma cell panel (*n* = 9) for in vitro analyses. We investigated altered gene expression, signaling events and *TERT* promoter activity upon BRAF- and E-twenty-six (ETS)-factor inhibition by qRT-PCR, chromatin immunoprecipitation (ChIP), Western blots and luciferase reporter assays. *TERT* promoter mutations were significantly enriched in *BRAF*^*V600E*^-mutated HGG as compared to *BRAF*^*V600E*^-mutated LGG. In vitro, *BRAF*^*V600E*^*/TERT* promoter double-mutant glioma cells showed exceptional sensitivity towards BRAF-targeting agents. Remarkably, BRAF-inhibition attenuated *TERT* expression and *TERT* promoter activity exclusively in double-mutant models, while *TERT* expression was undetectable in *BRAF*^*V600E*^-only cells. Various ETS-factors were broadly expressed, however, only ETS1 expression and phosphorylation were consistently downregulated following BRAF-inhibition. Knock-down experiments and ChIP corroborated the notion of a functional role for ETS1 and, accordingly, all double-mutant tumor cells were highly sensitive towards the ETS-factor inhibitor YK-4-279. In conclusion, our data suggest that concomitant *BRAF*^*V600E*^ and *TERT* promoter mutations synergistically support cancer cell proliferation and immortalization. ETS1 links these two driver alterations functionally and may represent a promising therapeutic target in this aggressive glioma subgroup.

**Electronic supplementary material:**

The online version of this article (10.1186/s40478-019-0775-6) contains supplementary material, which is available to authorized users.

## Introduction

Glioma represents the most common tumor type in the central nervous system (CNS) across all age groups [37]. The biology and clinical behavior of glioma are highly heterogeneous as reflected by WHO grades ranging from I to IV [27]. Generally, they are divided into low-grade glioma (LGG), comprised of WHO grades I/II, and WHO grade III/IV tumors which are referred to as high-grade glioma (HGG). Moreover, glioma encompasses a variety of histologic subtypes some of which can present either as LGG or HGG [27].

BRAF is a serine/threonine kinase and central mediator in the well-described oncogenic mitogen-activated protein kinase (MAPK) signaling pathway [14]. Various alterations such as activating mutations of *BRAF* are commonly found in cancerous tissues [14]. In the pediatric patient population, more than half of LGG are characterized by genetic alterations of the *BRAF* gene resulting in increased cellular proliferation due to hyperactivation of downstream signaling [16, 39]. Moreover, the missense mutation *BRAF*^*V600E*^ is present in a considerable amount of LGG namely pleomorphic xanthoastrocyma (PXA) and ganglioglioma (GG), but also other subtypes of astrocytoma [43]. With respect to HGG, *BRAF*^*V600E*^ has been described in anaplastic PXA or anaplastic GG [43], as well as pediatric (6–12%) [8, 43] and adult (7.7%) glioblastoma (GBM), often accompanied by an epithelioid phenotype [8, 20]. The biological differences between *BRAF*-mutant LGG and HGG remain poorly understood. To date, only concomitant deletion of the *CDKN2A* locus has been described to synergistically promote glioma development [15] and to define inferior outcome in *BRAF*^*V600E*^-positive glioma [21, 34]. Small-molecule inhibitors of BRAF and its downstream-target MEK have already been approved for other BRAF-driven cancer types, such as melanoma [14] and have been shown to effectively inhibit glioma growth both in preclinical models [5, 9, 22, 36] and small patient cohorts [7, 17, 21]. Consequently, phase I/II trials with BRAF- or MEK-inhibitors either as single agent (NCT01677741, NCT01748149, NCT03363217, NCT01089101, NCT02285439, NCT03213691) or in combination (NCT02684058, NCT03340506, NCT02034110) have already been initiated. First analyses show promising results in both the pediatric [3, 7, 21, 42] and the adult patient population [17].

The *telomerase reverse transcriptase (TERT)* gene codes for the core catalytic subunit of telomerase, an enzyme which is responsible for elongating the telomeric ends of chromosomes, thereby enabling cancer cells to bypass senescence. Hence, telomerase re-activation is a frequent mechanism, used in malignant tissues to render replicative immortality and is associated with worse prognosis in various types of brain tumors [11, 26]. Specific mutations within the *TERT* promoter, C250T (−146C > T), C228T (−124C > T) and A161C (−57A > C), have been identified to play an important role in telomerase re-activation in multiple tumor types including HGG [13, 46]. C228T represents the most frequent of either mutation in both LGG as well as HGG [18]. Functionally, all three non-coding mutations open new binding-sites for e-twenty-six (ETS/TCF) family transcription factors involved in *TERT* promoter hyperactivation [4, 13]. In addition to a major role of GABPA [4], contribution of MAPK-activated ETS-factors have been reported in *BRAF*-mutant melanoma and thyroid cancer [45, 50].

Pathologic activation of the MAPK signaling pathway in cancer cells is well-known to cause oncogene induced senescence (OIS), a tumor suppressing mechanism [38] which has also been described in *BRAF-*altered glioma [2]. Interestingly, re-expression of *TERT* has been shown to promote escape from OIS in *BRAF*-mutant cancer cells [38]. Moreover, *TERT* promoter and *BRAF* double-mutant papillary thyroid cancer exhibits a particularly aggressive course of disease, suggesting an important interaction of these two prominent oncogenic genomic aberrations [35, 52]. In brain tumors, cases with concurrent mutations of *BRAF* and the *TERT* promoter have been identified and appear to be associated with an aggressive tumor biology [29, 33, 34, 40, 54].

Hence, in this study we sought to elucidate the role of concomitant *BRAF*^*V600E*^ and *TERT* promoter mutations in the malignant phenotype of glioma, to dissect the involvement of different ETS-factors and investigate potential therapeutic implications.

## Materials and methods

### Clinical samples and patient data

Tumor tissues for analyses and establishment of patient-derived cell models were derived from patients treated at the General Hospital of Vienna or the Department of Neurosurgery at the Neuromed Campus, Kepler University Hospital in Linz. The histopathological diagnoses were assessed by experienced neuropathologist teams according to the 2016 WHO classification. Clinical histories and characteristics were obtained from patient charts available at the respective hospitals.

### Cell culture

All cell models were kept under humidified conditions containing 5% CO2 at 37 °C (normal cell culture conditions) and were regularly checked for mycoplasma contamination. Cell authentication was performed by short tandem repeat (STR) analysis. All primary glioma cell lines originating from the Department of Neurosurgery, Neuromed Campus, Kepler University Hospital, Linz (BTL53, BTL1333, BTL1304, BTL2231, BTL2176) and from the Medical University of Vienna (VBT4, VBT92, VBT125, VBT150, VBT172) were cultured in RPMI-1640 medium (Sigma-Aldrich, Missouri, USA) supplemented with 10% fetal calf serum (FCS, Gibco, Thermo Fisher Scientific, MA, USA).

NMC-G1, and AM38 cells were purchased from the Japanese Collection of Research Bioresources Cell Bank (Japan) and were cultured according to the distributor’s recommendations. DBTRG-05MG was purchased from the “Deutsche Sammlung von Mikroorganismen und Zellkulturen GmbH” (Braunschweig, Germany) and cultured in RPMI-1640 medium supplemented with 10% FCS. Neither antibiotics nor any other anti-microbial substances were used during this study. All experiments with both primary and stable cell models were performed between passages 5 and 15.

### Molecular characterization

DNA of tumor tissues and cell cultures was extracted and characterized for the respective *BRAF*^*V600E*^ and *TERT* promoter mutation status by direct sequencing as previously published [47]. For international cell models, available genetic information was extracted from the COSMIC database [48]. *CDKN2A* status was assessed by Ion Torrent sequencing and qRT-PCR, whilst activation of CDK4/6-signaling was estimated by detecting the phosphorylation of the Retinoblastoma-associated protein (Rb) on immunoblots. Copy number variants of the *CDNK2A* and *TERT* locus were confirmed using array comparative genome hybridization data either derived from COSMIC database (NMC-G1, DBTRG-05MG, AM38) [48] or analyzed in house as previously published (BTL1333, BTL53, BTL2176, VBT92, VBT125) [32]. The p53-pathway was evaluated through expression analysis of total p53 and the downstream target p21 by Western blot as well as sequencing data from COSMIC database (NMC-G1, DBTRG-05MG, AM38) [48] or established by Ion Torrent sequencing (BTL53, BTL1333, BTL2176, BTL2231, VBT92, VBT125, BTL1304). The Ion Torrent PGM System, the “Ion AmpliSeq Cancer Hotspot Panel v2 with 207 Amplicons” library and “Ion Torrent Suite Software (Version 5.10.1)” software were used to sequence tumor hotspot mutations. Sequencing data from NMC-G1, DBTRG-05MG and AM38 were derived from COSMIC, however, only previously reported pathogenic mutations were included in the manuscript.

### In silico analyses

RNA sequencing data derived from the cancer genome atlas (TCGA) from GBM (*n* = 166), skin cutaneous melanoma (*n* = 469) and bladder urothelial carcinoma (*n* = 408) were stratified according to their *BRAF* mutation status. Average logarithmic relative expression values of *TERT* and different ETS-factors in the respective subgroups were calculated by RNA sequencing algorithms (DESeq2). A dataset of glioma cases including information on *BRAF* and *TERT* promoter status, WHO grade and histologic subtype was compiled from COSMIC [48] database and a previously published dataset [18].

### Colony formation assay

Dabrafenib, vemurafenib, and YK-4-279 were purchased from Selleck Chemicals (Houston, TX, USA). Low densities of cells ranging from 0.5 × 10^3^-3 × 10^3^ cells/well depending on the respective cell proliferation time were seeded in 500 μl growth media in duplicates in 24-well plates and settled for 24 h under normal cell culture conditions. Upon the recovery time, the indicated drug concentration was added in 100 μl growth medium. Following drug exposure time of 7 days, cells were washed with phosphate-buffered saline (PBS) and fixed with ice-cold methanol for 30 min at 4 °C before cells were stained using crystal violet. Digital photographs were taken using a Nikon D3200 camera and processed with ImageJ software. For quantification, crystal violet was eluted using 2% sodium dodecyl sulfate and color absorbance was measured at 560 nm at the Tecan infinite 200Pro (Zurich, Switzerland). Values were analyzed using GraphPad Prism software 5.0 and are given in arbitrary units (AU) as mean +/− standard deviation (SD) normalized to untreated control.

### Cell viability assay – ATP assay

Dabrafenib, vemurafenib, and YK-4-279 were purchased from Selleck Chemicals (Houston, TX, USA). Cells were seeded in 100 μl of the respective growth medium in triplicates of 96-well plates at cell densities ranging from 2 × 10^3^-4 × 10^3^/well cells depending on the respective cell proliferation time. Following a 24 h recovery time under normal cell culture conditions, cells were treated with different drug concentrations in 100 μl growth medium. Upon 72 h, cell viability was analyzed based on the cellular ATP content following manufacturer’s instructions (“CellTiter-Glo® Luminescent Cell Viability Assay”, Promega, Madison, WI, USA). Luminescence was measured at 1000 nm at the Tecan infinite 200Pro. Raw data were analyzed using GraphPad Prism software 5.0. Results are given as mean +/− SD and were normalized to untreated control cells.

### Protein isolation and Western blotting

4 × 10^5^-6 × 10^5^ cells/well were seeded in 2 ml of growth medium in 6-well plates and left under normal cell culture conditions for recovery. On the next day when 80–90% confluence was reached, cells were treated with 1 μM dabrafenib for 6 h. Upon scraping and washing the cells in PBS, cells were mechanically (ultrasound) and chemically lysed (lysis buffer: 50 mM Tris/HCl (pH 7.6), 300 mM NaCl, 0.5% Triton X-100, supplemented with protease inhibitors PMSF and complete and phosphatase inhibitor PhosSTOP; all supplements from Roche, Rotkreuz, Switzerland).

Total protein concentrations were determined following manufacturer’s instructions (“Pierce™ BCA Protein Assay Kit”, Rockford, IL, USA). 15 μg of proteins were loaded onto 10% polyacrylamide-gels and polyacrylamide gel electrophoresis was run at 90 V. Proteins were blotted onto polyvinylidenfluorid membranes via semidry Western blotting. Blotting efficiency was checked with Ponceau protein staining. Antibodies detecting target proteins (Additional file 1: Table S1) were diluted 1:1000 in 3% BSA in Tris-buffered saline with 0.1% Tween20.

### RNA-extraction, reverse transcription and qRT-PCR

4 × 10^5^-6 × 10^5^ cells/well were seeded in 6-well plates in 2 ml of the respective growth medium. Upon 1 day, cells were exposed to 1 μM dabrafenib for 16 h. Total RNA was isolated using TRIzol reagent (Thermo Fisher Scientific, Waltham, MS, USA) and chloroform isolation according to standard protocols and checked for purity (260/280 ratio > 1.8) and concentration (100-500 ng/μl) using Nanodrop 1000 (Thermo Fisher Scientific). 1 μg of RNA was reverse transcribed into cDNA using Revert aid reverse transcriptase (Thermo Fisher Scientific). cDNA was diluted 1:25 and mixed to the same parts with 2x GoTaq Green Master Mix (Promega) and 10 nM of both forward and reverse primers (Eurofins Scientific, Luxembourg, Luxembourg; primer table in Additional file 1: Table S2). CFX Connect Real-Time PCR Detection System and analysis software (BioRad, Hercules, CA, USA) was used for running quantitative PCR. Raw data were normalized to internal control *RPL-41* (dCT) and converted to a linear form using 2^-dCT^ (mean from triplicates). In treatment experiments, expression values were normalized to the housekeeping gene *RPL-41* as well as to the respective untreated control (ΔΔCT), set as 1, and were converted to a linear form using 2^-ΔΔCT^ (mean, +/− SEM from triplicates).

### siRNA-mediated knock-down of ETS1

2 × 10^5^-3 × 10^5^ cells/ml were seeded in 500 μl or 2 ml growth medium into 24-well or 6-well plates, respectively, and incubated for 24 h under standard  cell culture conditions in order to recover. On the following day, knock-down was performed using 50 nM ETS1-targeting SMARTpool siRNA (UCAUUAGCUAUGGUAUUGA, GUCUCAAGCAUUAAAAGCU, CCCCAAGGUUUAAAUACAA, GGUUGGACUCUGAAUUUUG) or 50 nM Accell Green non-targeting siRNA (GE Healthcare Little Chalfont, UK). Transfection was performed using Xfect RNA transfection reagent (Takara Bio, Kyoto, Japan) according to company’s recommendations. Upon 48 h of incubation under normal cell culture conditions, total RNA was isolated and qRT-PCR was performed as described above.

### Luciferase reporter assay

4 × 10^5^-6 × 10^5^ cells were seeded in 2 ml of growth medium into 6-well plates and incubated under standard cell culture conditions for 24 h. Upon recovery, cells were transfected with the indicated plasmids as described previously [47] using Lipofectamine 3000 according to manufacturer’s recommendations. After incubation for 24 h, cells were treated with 1 μM dabrafenib. Following 16 h drug exposure, proteins were isolated and luciferase signals were analyzed using the Dual-Glo Luciferase Assay System (Promega) according to manufacturer’s instructions.

### ChIP

Protein crosslinking was performed using 1% methanol-free paraformaldehyde (Thermo Fisher Scientific) and reaction was stopped with glycine. Dynabeads Protein A (Thermo Fisher Scientific) were precleared, subsequently blocked using bovine serum albumin and finally loaded with antibodies targeting ETS1, GABPA, IgG and AcH3K27 described in Additional file 1: Table S1. Chromatin was sonicated and validated for suitable fragment size via agarose gels. Crosslinked protein-chromatin suspension was added to the antibody pre-loaded beads and incubated over night at 4 °C on an overhead rotator. The next day, beads were washed to remove unbound fragments and DNA was eluted from the beads upon heat-induced reverse-crosslinking. DNA was isolated by phenol-chloroform (Sigma Aldrich) purification and genomic fragments were quantified with qRT-PCR as described above using primers adjacent to the prominent *TERT* promoter mutations C228T and C250T. The used primer sequences are listed in Additional file 1: Table S2.

### Ectopic *TERT* expression using adenoviral constructs

The HA-tagged TERT adenoviral construct (HA-TERT, human, #349917A) was purchased from ABM (Richmond, BC, CAN) and multiplied by several rounds of HEK-293 cell amplification. GFP adenovirus was constructed using AdEasy Adenoviral Vector System (Agilent, La Jolla, CA, USA) and served as infection control. Cells were cracked by three freeze and thaw cycles in Tris/HCl (pH 8). DBTRG-05MG cells were infected using 30 moi of the respective viruses. RNA for qRT-PCR (primer sequences are listed in Additional file 1: Table S2) were isolated 48 h upon infection. For viability test by ATP-assay, cells were counted and seeded 48 h after virus infection. 24 h later, cells were treated with YK-4-279 and cell viability was measured after 72 h (see cell viability assay section above).

## Results

### *TERT* promoter mutations are associated with *TERT* expression and enhanced aggressiveness in *BRAF*^*V600E*^-mutated glioma

We analyzed the *TERT* promoter mutation status in a small cohort of pediatric cases with *BRAF*^*V600E*^-mutated glioma (*n* = 8, Additional file 1: Table S3) treated at the General Hospital of Vienna. *TERT* promoter mutation status of these *BRAF*^*V600E*^-positive glioma patients was correlated to clinical parameters including gender, age, WHO grade and overall survival. Interestingly, the single patient harboring a tumor with additional *TERT* promoter mutation showed the most aggressive course of disease (Additional file 1: Table S3). To further analyze this clinical finding on a broader basis, we curated a dataset of 103 *BRAF*^*V600E*^-mutated glioma with information on tumor grade and *TERT* promoter mutation from publicly available datasets (Additional file 2: Table S4). Corroboratively, double-mutant tumors were significantly enriched (Fisher’s exact test, *p* = 0.003) in HGG (WHO grade III/IV; 19/69, 28%) as compared to LGG (WHO grade I/II, 1/34, 3%).

To investigate the underlying oncogenic mechanisms of concomitant *TERT* promoter and *BRAF*^*V600E*^ mutations in glioma, we analyzed the expression of different ETS-factors (*ETS1, GABPA, GABPB-1S, GABPB-1 L, GABPB-2*) and their downstream targets c*yclin D1* and *TERT* in tissues of *BRAF*^*V600E*^-positive glioma (n = 8) (Additional file 3: Figure S1). Strikingly, *TERT* mRNA was only expressed in the tumor with concomitant *TERT* promoter and *BRAF*^*V600E*^ mutations, whereas neither differences in expression of ETS-factors nor the downstream target c*yclin D1* were observed (Additional file 3: Figure S1). This was confirmed by analysis of in silico RNA sequencing data, additionally including the ETS-factors ETV1, ETV4 and ETV5 (Additional file 3: Figure S2A). Interestingly, investigation of in silico data rather showed a trend towards lower *TERT* mRNA expression in *BRAF*-mutant GBM, but not other *BRAF*-mutant tumor types (Additional file 3: Figure S2B).

Based on these data, we sought to elucidate the interplay of BRAF^V600E^ signaling and downstream effects on the *TERT* promoter in more detail. Therefore, we established a panel of twelve glioma-derived cell lines with different *BRAF* and *TERT* promoter status, containing nine *BRAF*^*V600E*^-mutant and three *BRAF* wild-type cell models (Table 1). The latter were considered as references to dissect the effect of oncogenic BRAF activation in the background of both a mutated (BTL2176) and a wild-type (BTL1333, BTL53) *TERT* promoter. Consistent with our previous tissue analyses, only *BRAF*^*V600E*^/*TERT* promoter-mutant cell lines, but not the models with an isolated *BRAF*^*V600E*^ mutation, expressed *TERT* mRNA (Table 1). Notably, all available *BRAF*^*V600E*^-positive stable cell lines derived from our neurosurgical departments or from commercial sources turned out to be *TERT* promoter-mutated. In contrast, primo-cell cultures from three *BRAF*^*V600E*^-positive glioma specimens not expressing *TERT* mRNA and not developing into stable cell models were *TERT* promoter-wild-type (Table 1). Consequently, only one of these *BRAF*^*V600E*^*/TERT* promoter wild-type primo-cell models could be propagated sufficiently for further in vitro experiments. Moreover, none of the double-mutant cell lines showed a gain of the *TERT* gene locus (Additional file 3: Figure S3), supporting our hypothesis that promoter mutation is the primary driver of telomerase re-activation in these tumors.

**Table 1:** Histopathological and molecular characteristics of the cell models

	Histology	*BRAF* V600E	*TERT* prom. mutation	*TERT* mRNA expression	CDKN2A expression	Additional genetic aberrations	Stable cell line
BTL1333	GBM^*^	wt	wt	neg	pos	TP53(D228V*)	yes
BTL53	GBM	wt	wt	pos	pos	*TP53(V173M)* *RB1 deletion*	yes
BTL2176	GBM	wt	C228T	pos	neg	*PIK3CA(N1044K)*	yes
NMC-G1	GBM	pos	C228T (homozygous)	pos	neg	*–*	yes
DBTRG-05MG	GBM	pos	C228T	pos	neg	POT1(G40*)	yes
AM38	GBM	pos	C250T	pos	neg	*ALK(S737 L)*	yes
VBT92	aPXA^+^	pos	C228T	pos	neg	*–*	yes
VBT125	GS^o^	pos	C228T	pos	neg	*–*	yes
BTL1304	GS	pos	C228T	pos	neg	*PTEN(K266E)*	yes
BTL2231	PXA^#^	pos	wt	neg	neg	–	no
VBT150	PXA	pos	wt	neg	pos	n.a.	no
VBT172	aPXA	pos	wt	neg	pos	n.a	no

^*^ GBM = glioblastoma multiforme

^+^aPXA = anaplastic pleomorphic xanthoastrocytoma

^o^GS = gliosarcoma

^#^PXA = pleomorphic xanthoastrocytoma

*pos* positive, *neg* negative, *wt* wild-type, *mut* mutated, *n.a.* not analyzed

We further characterized the panel for the respective *CDKN2A* and *TP53* status. All analyzed *BRAF*^*V600E*^ cell models lacked *TP53* mutations and, in contrast to *BRAF* wild-type cells, homogenously expressed the p53 downstream target p21 (Additional file 3: Figure S3). Notably, only tumor cell explants with a *BRAF*^*V600E*^-mutated background harboring loss of *CDKN2A* expression in combination with *TERT* promoter mutation developed into stable, immortalized cell lines (Table 1, Additional file 3: Figure S3). In line with CDKN2A loss-of-function and consecutive activation of the cyclin D1/cyclin dependent kinase (CDK) 4/6 complex [15], Rb was hyperphosphorylated in the majority of double-mutant glioma cells, indicating functional inhibition (Additional file 3: Figure S3).

### ETS-factors are hyperactivated in *BRAF*^*V600E*^-mutated glioma cells

**Fig. 1:**
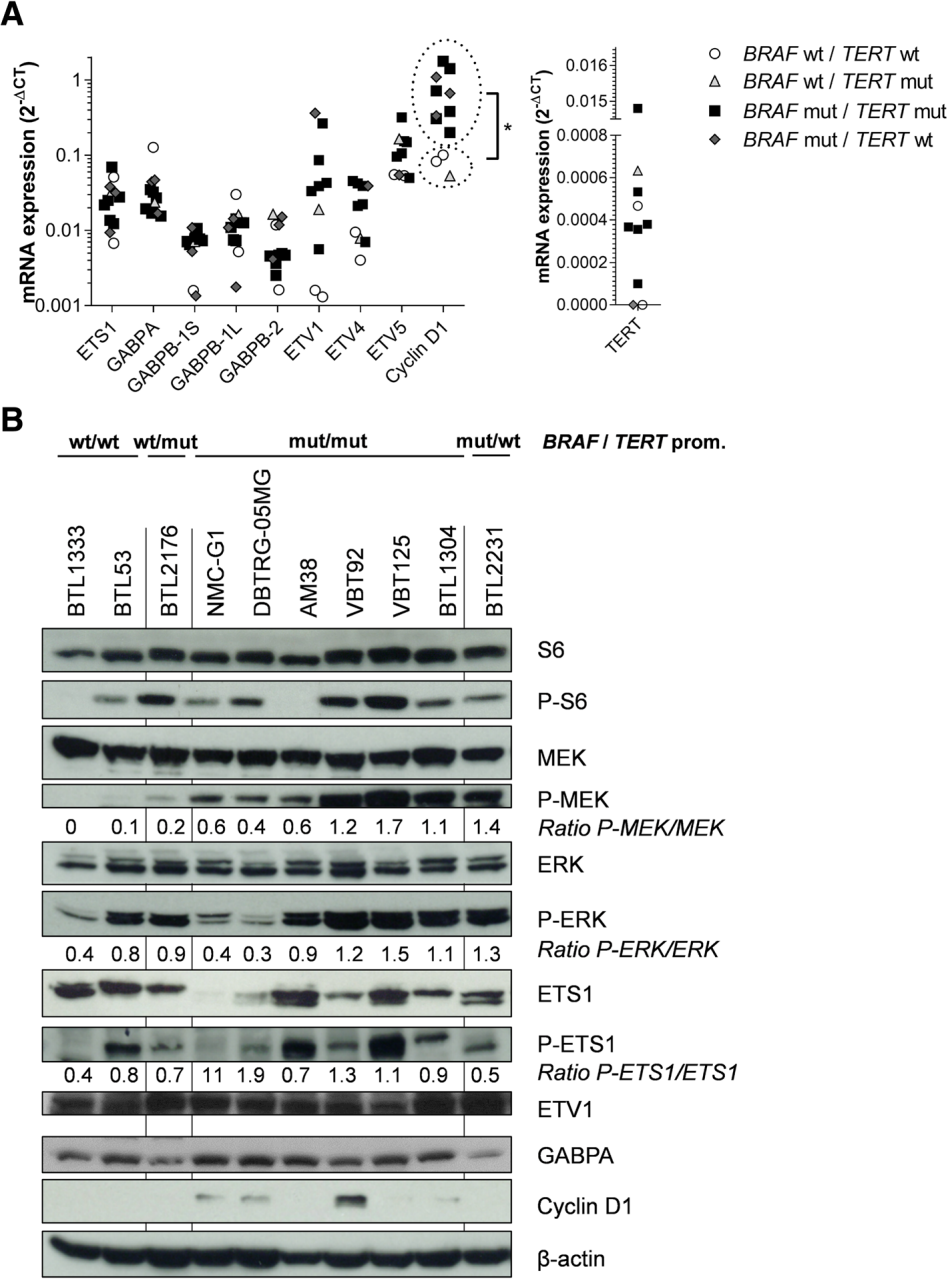
Expression patterns of *TERT*, ETS-factors and activation of associated signaling cascades. **a** mRNA expression of ETS-factors and the ETS-downstream targets c*yclin D1* and *TERT* were analyzed in the indicated genotypes. Means of three independent experiments are shown. Cyclin D1 mRNA expression of *BRAF* wild-type versus *BRAF*^*V600E*^ mutated cell lines was quantified by unpaired student’s t-test (*p < 0.05). **b** Western blot analyses of cell lines with different *BRAF* and *TERT* promoter status as indicated are depicted. Proteins of S6 and MAPK pathway as well as selected ETS-factors and downstream targets are shown. Ratios between phosphorylated and total proteins as indicated were calculated after normalization to β-actin. wt = wild-type, mut = mutated

Based on the well-described activation of ETS-factors via MAPK-signaling [51] and having confirmed ETS-factor expression in the respective tumor tissues, we hypothesized that *BRAF*^*V600E*^ mutant glioma cells were characterized by hyperactivated ETS-signaling. Analyses of ETS-factor mRNA expression confirmed that the transcription factors *ETS1*, *GABPA, GABPB, ETV1, ETV4 and ETV5* were widely expressed throughout the entire cell panel, however, no differences between the genotypes were observed. Similarly, the distinct splice-variants of *GABPB* (*GABPB-1S, GABPB-1 L, GABPB-2*) showed no differences in expression between the genotypes. In contrast, *cyclin D1* displayed significantly higher expression in *BRAF*^*V600E*^ positive cell models as compared to wild-type cells. No differences between the investigated genotypes were observed for *TERT* mRNA expression (Fig. 1a).

Next, we investigated expression and signaling activation of MAPK pathway members on protein level. Consistent with oncogenic BRAF signaling, phosphorylation levels of MEK were markedly enhanced in *BRAF*^*V600E*^-mutant as compared to *BRAF* wild-type cell models. In contrast, no distinct differences were observed for activation of S6 and ERK indicating activation of the respective pathways by alternative mechanisms in the *BRAF* wild-type models, an effect which has previously been described in both glioma [36] and melanoma [55]. With respect to ETS-factors, ETS1 was variably expressed throughout the cell panel and showed phosphorylation in all genotypes (Fig. 1b, Additional file 3: Figure S4). GABPA was widely and ETV1 constitutively expressed in the investigated cell line panel. In line with our qRT-PCR results, cyclin D1 was predominantly detectable in cell models harboring *BRAF* alterations (Fig. 1b).

### *BRAF*^*V600E*^/*TERT* promoter double-mutant glioma cells are highly sensitive towards BRAF-inhibitors

**Fig. 2:**
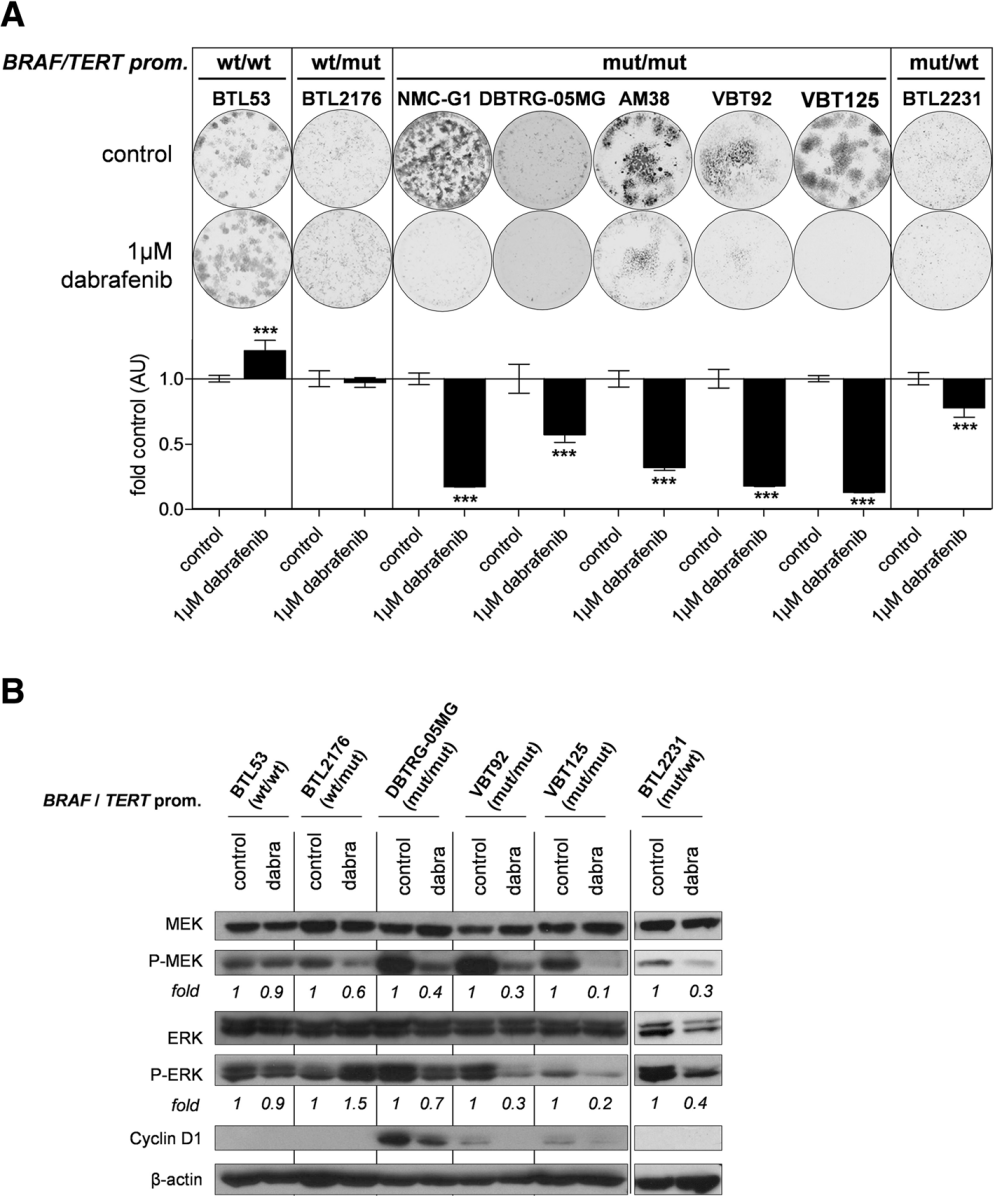
Anti-proliferative effects and altered downstream-signaling upon BRAF-inhibition. **a** Clone formation assays of with different *BRAF* and *TERT* promoter status as indicated are shown. Cells were seeded at low density and treated with 1 μM dabrafenib for 7 days. The upper panel depicts one representative well per condition. The lower panel shows the quantitative results represented as mean +/− SD of the respective untreated control. ***p < 0.001 (unpaired student’s t-tests) (**b**) Western blot analyses of cell models with different *BRAF* and *TERT* promoter status are depicted. Cell models were treated with 1 μM dabrafenib for 6 h. Expression and phosphorylation of the indicated MAPK pathway mediators as well as cyclin D1 are shown. Fold values are given as normalized expression to β-actin followed by activated kinase/total kinase and are normalized to the respective control. wt = wild-type, mut = mutated, dabra = dabrafenib

In order to validate the expected dependency of *BRAF*^*V600E*^-mutant glioma on hyperactivated MAPK-signaling, we tested the anti-proliferative effects of the BRAF-inhibitor dabrafenib. As predicted, dabrafenib was only effective in *BRAF*-mutated cell models (Additional file 3: Figure S5a, Fig. 2a). Interestingly, sensitivity was highest in those cell lines harboring additional *TERT* promoter mutations (Fig. 2a). In contrast, cell proliferation of cell models lacking *BRAF*^*V600E*^ mutation was not inhibited but rather increased (Fig. 2a). In addition, these results were confirmed with vemurafenib, another BRAF-inhibitor (Additional file 3: Figure S5b). In addition to basal expression levels, we further investigated downstream effects of BRAF-inhibition in the respective genotypes. Dabrafenib effectively decreased both MEK- as well as ERK-phosphorylation levels in all *BRAF*^*V600E*^-mutated cell lines. In contrast, despite inhibition of MEK-phosphorylation, ERK-phosphorylation was stable or even increased in *BRAF* wild-type cell models (Fig. 2b). This finding is well in agreement with the so-called RAF-paradox by BRAF-inhibition under wild-type conditions [14]. Moreover, dabrafenib treatment resulted in downregulation of cyclin D1 in double-mutant cell models (Fig. 2b). No effect on cell proliferation was observed upon short term BRAF-inhibitor treatment (data not shown).

### Oncogenic MAPK-signaling mediates *TERT* expression in *BRAF*^*V600E*^*/TERT* promoter double-mutant glioma

**Fig. 3:**
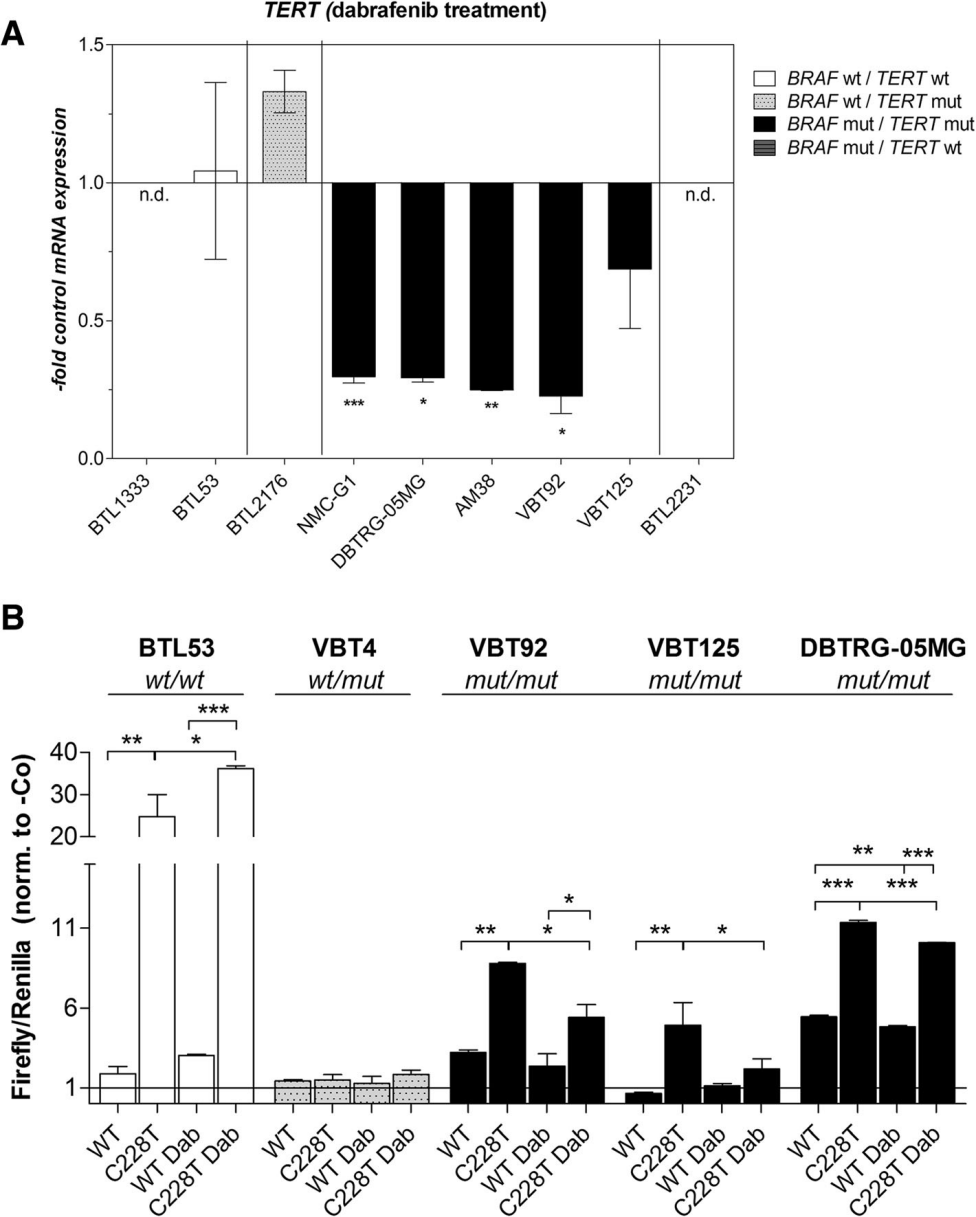
Regulation of *TERT* expression and *TERT* promoter activity upon BRAF-inhibition. **a** *TERT* mRNA expression following dabrafenib treatment (1 μM, 16 h) of the indicated cell models is shown. Mean +/− SD; unpaired student’s t-tests (**b**) Luciferase reporter assays were performed in cell lines with different *BRAF* and *TERT* promoter status as indicated using wild-type or mutated (C228T) *TERT* promoter sequences. Cells were treated with 1 μM dabrafenib for 16 h. Results are given as ratio of firefly to renilla luciferase (internal control) and were normalized to a promoter-less construct (−Co, set to 1). Values are given as mean +/− SD from duplicates. One representative experiment out of three, delivering comparable results, is shown. Tukey’s multi-comparison one-way ANOVA was applied for statistical analysis. *p < 0.05, **p < 0.01, ***p < 0.001; wt = wild-type, mut = mutated, n.d. = not detected, dab = dabrafenib

After confirming the respective impact of BRAF-inhibition on downstream signaling in the different genotypes, we tested the effect of dabrafenib on *TERT* expression. *TERT* mRNA was constitutively downregulated by dabrafenib (Fig. 3a) or vemurafenib (Additional file 3: Figure S6) in double-mutant glioma models only. Conversely, dabrafenib rather stimulated *TERT* expression in *BRAF* wild-type/*TERT* promoter-mutant cells and had no effect in the double wild-type background. No effect on cell proliferation was observed upon short term BRAF-inhibitor treatment (data not shown). In order to clarify the role of *TERT* promoter mutation in telomerase re-activation in the respective genotypes, we analyzed activation of the wild-type and mutant promoter sequences in each genotype via luciferase reporter assays. As expected, the C228T-mutated *TERT* promoter construct was significantly more active as compared to the wild-type promoter in most of the cell models. This high promoter activity could be suppressed by treatment with dabrafenib solely in the *BRAF*^*V600E*^-mutated cell models. In contrast, BRAF-inhibition increased the activity of the mutated *TERT* promoter in a double wild-type background (Fig. 3b).

### ETS1 activation is impaired upon BRAF-inhibition in *BRAF*^*V600E*^*-*mutant glioma

**Fig. 4:**
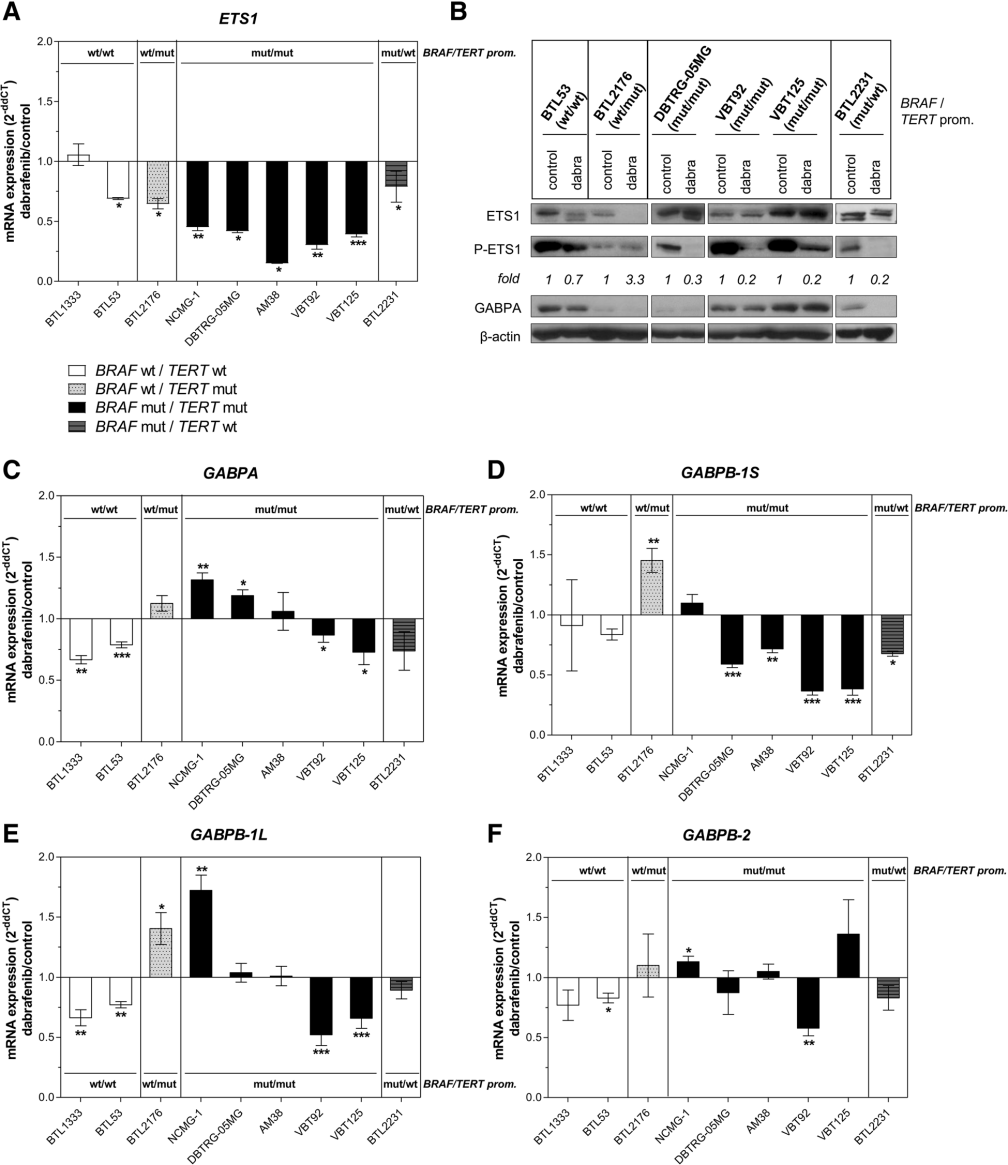
Regulation of ETS-factors by oncogenic BRAF signaling. **a** mRNA and (**b**) protein expression/phosphorylation levels of ETS1/GABPA upon 16 h at 1 μM (qRT-PCR) and 6 h at 1 μM (Western blot) dabrafenib treatment. Fold values are given as normalized expression to β-actin and subsequent calculation of the ratio phospho/total ETS1 and are normalized to the respective untreated controls. mRNA expression levels of (**c**) *GABPA,* (**d**) *GABPB-1S,* (**e**) *GABPB-1 L,* and (**f**) *GABPB-2* are depicted for the indicated cell models upon dabrafenib treatment (1 μM, 16 h). *p < 0.05, **p < 0.01, ***p < 0.001 (unpaired students’ t-tests); All values are given as mean +/− SD; wt = wild-type, mut = mutated, dabra = dabrafenib

In order to investigate the impact of BRAF-mediated downstream signaling on ETS-factors in more detail, we selected ETS1, which is well described for being transcriptionally and post-translationally activated by MAPK signaling, for further analyses [30, 44, 50]. In correspondence to the expression patterns observed for *TERT, ETS1* was more effectively downregulated by dabrafenib in all *BRAF*^*V600E*^*/TERT* promoter-mutant cell models (Fig. 4a). Moreover, ETS1 activation via phosphorylation was blocked in response to the BRAF-inhibitors dabrafenib (Fig. 4b) and vemurafenib (Additional file 3: Figure S7) in all *BRAF*^*V600E*^-mutant cells hence paralleling phosphorylation of ERK. In contrast, the levels of ETS1 expression and phosphorylation in *BRAF* wild-type cell lines were not efficiently blocked, obviously based on the above described paradoxical ERK-activation by the BRAF-inhibitors under wild-type conditions. GABPA protein expression, however, was not affected by BRAF-inhibition in double-mutant glioma cells (Fig. 4b). To exclude whether this was a side-effect of cell growth inhibition we tested short-term BRAF-inhibitor treatment and observed no effect on cell proliferation (data not shown).

With respect to other ETS-factors, only *GABPB-1S* expression was also predominately inhibited by dabrafenib in a *BRAF*^*V600E*^-mutated background whereas *GABPA, GABPB-1 L* and *GABPB-2* showed variable response patterns (Fig. 4c-f). In line with the data from our cell models, a residual tumor of an anaplastic PXA case operated during combination treatment of dabrafenib and the MEK-inhibitor trametinib had lost expression of *ETS1*, *cyclin D1* and *TERT* (Additional file 3: Figure S8).

### ETS1 mediates *TERT* expression in *BRAF*^*V600E*^ and *TERT* promoter-mutated glioma cells

**Fig. 5:**
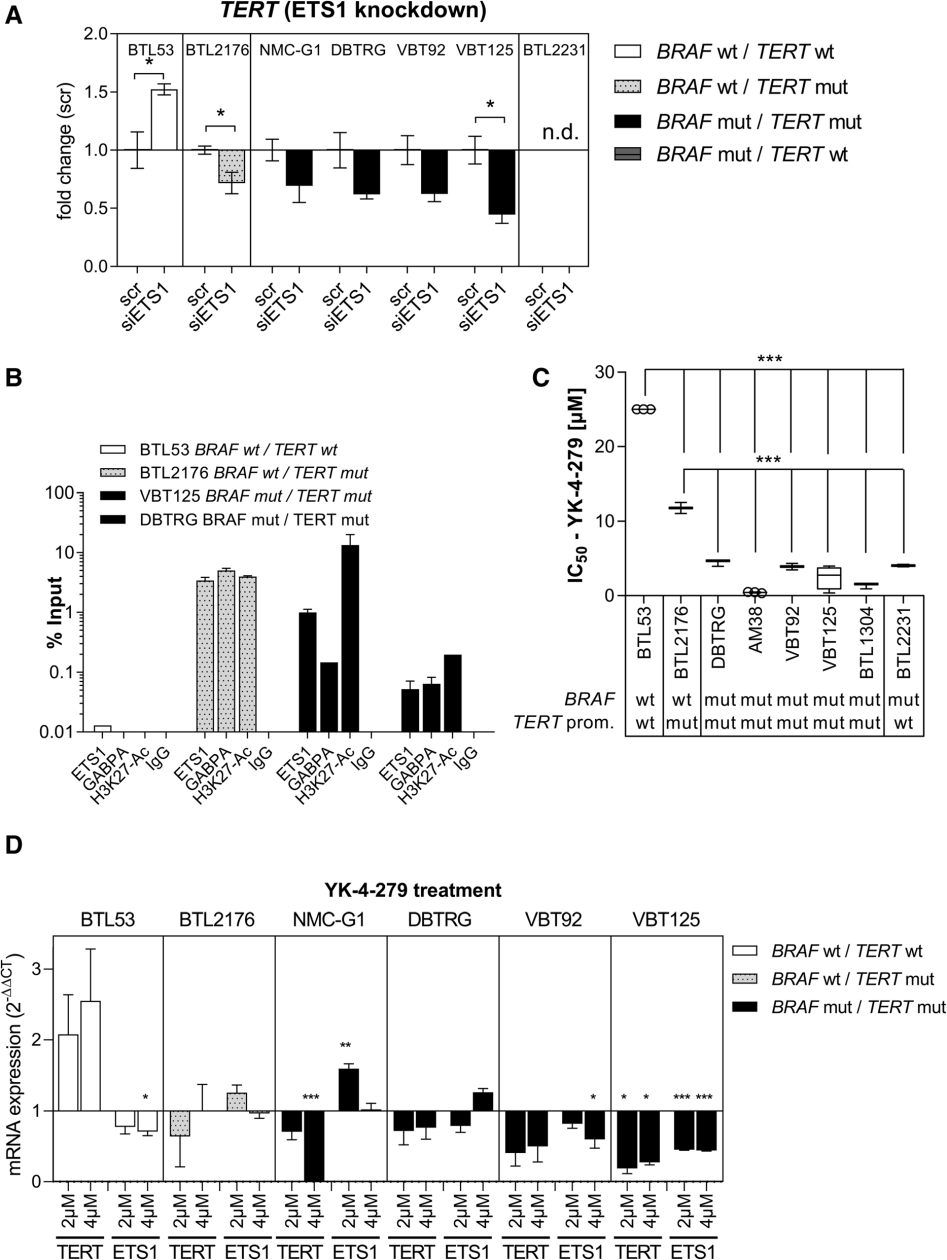
Activation of the mutant *TERT* promoter by ETS1. **a** An siRNA approach was applied to *ETS1* knock-down in cell models with different *BRAF* and *TERT* promoter status as indicated. *TERT* mRNA expression was detected using qRT-PCR. Significance levels were evaluated by unpaired students’ t-tests (mean +/− SEM). **b** Binding of ETS1 and GABPA to the different TERT promoter variants and presence of the activating histone mark H3K27-Ac was analyzed by ChIP-qRT-PCR. IgG served as negative control. Values are given as % Input, depicted mean +/− SD from duplicates. **c** Cytotoxicity assay were performed in cell models of different *BRAF* and *TERT* promoter background as depicted. Half-maximal inhibitory concentration (IC50) after YK-4-279 treatment for 72 h was calculated. Tukey’s multi-comparison one-way ANOVA was applied (mean +/− SD). **d** *TERT* and *ETS1* mRNA expression levels were analyzed by qRT-PCR after 16 h treatment with the indicated concentrations of YK-4-279. Ordinary one-way ANOVAs for every cell line were calculated (Dunnet correction, 0.05 (95% confidence interval) as controls vs. treatments (mean +/− SEM). *p < 0.05, **p < 0.01, ***p < 0.001, wt = wild-type, mut = mutated, n.d. = not detected

To more specifically dissect the link between MAPK-signaling and telomerase re-activation, we applied siRNA mediated knock-down of *ETS1*. Knock-down consistently decreased *TERT* mRNA expression across *BRAF*^*V600E*^*/TERT* promoter-mutated glioma cells as well as in a *TERT* promoter-mutated cell model (Fig. 5a). Notably, no effect on cell proliferation was observed upon siRNA-mediated knock-down (data not shown). Moreover, by ChIP-qRT-PCR we confirmed strong binding of ETS1 and GABPA selectively to the mutant *TERT* promoter locus, paralleled by activating H3K27-acetylation. In one double mutant cell model ETS1 even was the dominant of the investigated ETS-factors bound to the mutant promoter site (Fig. 5b). To clarify, whether ETS-factors play a central role in the malignant phenotype of *BRAF*^*V600E*^-mutant glioma, we assessed sensitivity to the ETS-inhibitor YK-4-279 [10] across our cell panel. Indeed, *BRAF-*mutant cell models were hypersensitive (IC_50_ < 5 μM) towards this compound suggesting a wide dependency of cell viability/proliferation on ETS-mediated signals (Fig. 5c). Accordingly, in contrast to *TERT* promoter wild-type glioma, ETS-factor inhibition by YK-4-279 reduced *TERT* mRNA expression in double-mutant cell models whereas ETS1 expression was more variable (Fig. 5d). Moreover, *TERT* re-expression partly rescued double-mutant glioma cells from the YK-4-279-induced growth inhibitory effect (Additional file 3: Figure S9). Combination of the BRAF inhibitor dabrafenib and the ETS-factor inhibitor YK-4-279 revealed additive to rather antagonistic effects, especially in the double-mutant glioma cell models (Additional file 3: Figure S10).

## Discussion

The discovery of genomic aberrations in *BRAF* as drivers of certain glioma subtypes has resulted in a magnificent extension of the therapeutic repertoire for these patients [17, 39, 43]. Recent studies, however, indicate that additional factors such as loss of *CDKN2A* or telomerase re-activation may significantly influence the clinical outcome of glioma patients with oncogenic *BRAF,* suggesting a biological heterogeneity within this subgroup [34, 40]. In this context, OIS and replicative senescence have been described as central obstacles for proliferation in *BRAF*-altered glioma cells [2]. Furthermore, *TERT* derepression has been demonstrated to promote escape of cancer cells from OIS [38]. Previous clinical observations and case-report studies delivered preliminary evidence that *TERT* promoter mutations indicate tumors with higher aggressiveness within *BRAF*^*V600E*^-mutated glioma [33, 34, 40, 54]. Consequently, we systematically investigated the cellular factors contributing to the interplay of *BRAF*^*V600E*^ and *TERT* promoter mutations in glioma.

To begin with, we analyzed the clinical impact of telomerase re-activation in a small cohort of *BRAF*^*V600E*^-mutated glioma. In accordance with previous reports, we observed a highly aggressive course in one anaplastic PXA with both *BRAF*^*V600E*^ and *TERT* promoter mutations in our patient collective [18, 28]. Moreover, only this double-mutant tumor expressed detectable levels of *TERT* mRNA. Consistent with this observation, an in silico analysis of 103 *BRAF*^*V600E*^-mutated glioma showed that *TERT* promoter mutations are significantly enriched in WHO grade III/IV tumors. Within this dataset, 28% of *BRAF*^*V600E*^-mutated HGG harbored additional *TERT* promoter mutations, which is consistent with earlier reports of secondary HGG and anaplastic PXA [18, 20, 28, 33, 34, 40]. Particular aggressiveness of double-mutant tumors has already been described for other tumor-types such as melanoma and thyroid cancer [23, 35, 52].

In order to elucidate the potential role of *TERT* promoter mutations in the malignant phenotype of certain *BRAF*^*V600E*^-mutated glioma, we curated a unique set of cell models containing nine *BRAF*^*V600E*^-mutant gliomas, the largest panel reported to date. Notably, all stable *BRAF*^*V600E*^-positive cell lines harbored *TERT* promoter mutations corroborating the aggressive biology of double-mutant tumors. Moreover, double-mutant models were further characterized by loss of the *CDKN2A* locus and BRAF-induced cyclin D1 expression. In a previous reported study, depletion of *CDKN2A*, *CDKN1A,* and *TP53* has been demonstrated as essential factor to overcome OIS in a patient-derived model of pilocytic astrocytoma [2]. These findings support our data as all stable *BRAF*^*V600E*^-mutant cell models harbor a loss at the *CDKN2A* locus. Moreover, the same study showed that *TERT* re-expression in this *BRAF*-driven cell model enabled escape from replicative senescence [2]. This supports the notion that the aggressive behavior of these tumors is fueled by a synergistic activation of MAPK-signaling in addition to CDK4/6-activation and telomerase re-expression.

The pathogenic mechanism of *TERT* promoter mutations is widely mediated by transcriptional activation which involves the binding of ETS-factors [13]. Specifically, the ETS-factors ETS1, GABPA, GABPB, and the splice variant GABPB-1 L, have been linked to activation of the mutated *TERT* promoter [4, 13, 31, 53]. Interestingly, both overall expression (e.g. *GABPB, ETS1*) as well as posttranscriptional activation (e.g. ETS1) can be stimulated via the MAPK-pathway [6, 25, 41, 51]. Accordingly, with respect to *BRAF*^*V600E*^-mutated melanoma, both phosphorylation of ETS1 and upregulation of GABPB via Fos have been shown to link oncogenic BRAF signaling to activation of the mutant *TERT* promoter [25, 50]. We analyzed the expression of the major ETS-factors *ETS1, GABPA*, and *GABPB* with its splice variants, ETV1, ETV4 and ETV5 all of which were widely expressed across our glioma cell panel. In contrast, *TERT* mRNA was only detectable in *BRAF*^*V600E*^ cells with additional *TERT* promoter mutations. Cell proliferation assays with BRAF-inhibitors confirmed the selective activity against *BRAF*^*V600E*^-mutated glioma cells, as already reported by previous preclinical studies and case series [7, 12, 19, 21, 22]. Strikingly, BRAF-inhibition also blocked activating phosphorylation of ETS1 selectively in *BRAF*^*V600E*^-mutant glioma cells. This effect has previously been described in other *BRAF*-mutated tumor types like melanoma [50], but not yet in glioma. Additionally, we could detect an inhibitory effect on cyclin D1 expression, confirming previous data, suggesting it as a downstream target of oncogenic MAPK-signaling in *BRAF*-mutated glioma [39].

In a following step, we demonstrated that activation of *TERT* transcription is dependent on BRAF signaling solely in the background of *BRAF*^*V600E*^ and *TERT* promoter double-mutation. Moreover, the clinical relevance of this finding is supported by investigation of the tumor material derived from a single patient treated with a combination of BRAF- and MEK-inhibitors resulting in undetectable levels of both *ETS1* and *TERT* during therapy. Oncogenic BRAF signaling has been shown to bridge telomerase re-activation via the mutated *TERT* promoter sequence. With respect to ETS-factor activation, *GABPB* expression has been described to be upregulated via Fos, a well described downstream effector of MAPK and ETS1 signals [25, 41], and ETS1 is directly phosphorylated via the MAPK-pathway [50]. Next, we tested the impact of BRAF-inhibition on the expression of the respective ETS-factors and found that only *ETS1* expression, but not expression of *GABPA* or the different *GABPB* splice variants, were significantly and consistently downregulated throughout all *BRAF*^*V600E*^-mutated glioma. These findings are well in agreement with a feed-forward loop on *ETS1* expression exerted by MAPK-signaling and ETS1 as predominant effector molecule [30]. Additionally, we show that knock-down of *ETS1* reduces *TERT* expression confirming it as mediator of *BRAF*^*V600E*^-driven *TERT* promoter activation. Accordingly, ChIP-analysis revealed ETS1 and GABPA-binding specifically to the mutant *TERT* promoter site. Previous studies in *BRAF* wild-type glioblastoma models have already demonstrated a central role of GABPA in activation of the mutant *TERT* promoter [4]. Our data point towards cooperation of GABPA with ETS1, especially in a *BRAF*^*V600E*^-mutant glioma background. Accordingly, a recent study in melanoma has shown that the C228T variant, the predominant mutation detected in our panel, is more efficiently activated by ETS1 as compared to the other *TERT* promoter mutations [1]. The potential role of other MAPK- induced ETS-factors is of high interest to be investigated in further studies.

BRAF-inhibition has become the standard of care in *BRAF*^*V600E*^-mutated melanoma [14]. However, acquired insensitivity plays an important role in therapy failure [24]. Additionally, first results of clinical application in *BRAF*^*V600E*^-positive glioma indicate that also a proportion of these tumors exhibit intrinsic BRAF-inhibitor resistance [17]. Consequently, inhibition of ETS-factors as downstream MAPK-signal transmitters have been suggested to provide a novel therapeutic opportunity to overcome upstream resistance development [49]. Therefore, we tested the effect of YK-4-279, a well-described ETS-factor inhibitor [10], in our cell line panel. In line with previous reports from our group concerning meningioma [47], also glioma cell models harboring mutant *TERT* promoters were hypersensitive towards YK-4-279 treatment [47] and YK-4-279 distinctly reduced *TERT* mRNA expression. Strikingly, *BRAF*^*V600E*^-mutant models were even more sensitive towards YK-4-279, irrespective of the underlying *TERT* promoter status. *TERT* re-expression from a viral promoter partially rescued double-mutated glioma cells from the cytotoxic effect of YK-4-279. As we found that both dabrafenib as well as YK-4-279 were highly active against *BRAF*^*V600E*^ mutated glioma, we aimed to investigate interactions between the two drugs. No synergistic, but rather antagonistic effects were identified particularly in the double-mutant glioma cell models. These results correspond to our findings that dabrafenib alone already reduced the expression levels of ETS1, the target of YK-4-279. Taken together, our data suggest that apart from the demonstrated *TERT* promoter activating properties, ETS-factors appear to play an important role in tumor biology of *BRAF*^*V600E*^-mutated glioma.

## Conclusions

Summarizing, we prove that telomerase re-activation based on a mutant *TERT* promoter sequence in *BRAF*^*V600E*^-mutant glioma is driven by oncogenic BRAF signaling predominantly via downstream activation of ETS-factors. Accordingly, ETS-factor inhibition is a promising therapeutic option for therapy-resistant *BRAF*-mutant glioma.

## Additional files

Additional file 1: **Table S1.** Antibodies. **Table S2.** Primers. **Table S3.** Histopathological, molecular and clinical parameters of the Vienna patient cohort. (DOCX 16 kb)

Additional file 2: **Table S4.** Dataset of 103 BRAFV600E-mutated glioma with information on tumor grade and TERT promoter mutation. (XLSX 10 kb)

Additional file 3: **Figure S1.** mRNA expression of ETS transcription factors and downstream targets in tumor tissue. **Figure S2.** Expression of Ets-factors and TERT in TCGA RNA sequencing data sets. **Figure S3.** Evaluation of TP53 and CDKN2A/Rb signaling pathways. **Figure S4.** Activation levels of MEK, ERK and ETS1. **Figure S5.** Anti-proliferative effects of BRAF inhibitors. **Figure S6.** Inhibition of TERT expression upon vemurafenib treatment. **Figure S7.** ETS1 inhibition upon vemurafenib treatment. **Figure S8.** Changes in expression patterns in patient tissue upon targeted treatment. **Figure S9.** Ectopic TERT re-expression partly rescues double-mutant glioma cells from YK-4-279- mediated cytotoxicity. **Figure S10.** Combined BRAF and Ets-factor inhibition. (PDF 786 kb)

## Data Availability

All data generated or analyzed during this study are included in this published article.

## Abbreviations

CDK: cyclin dependent kinase
ChIP: chromatin immunoprecipitation
CNS: central nervous system
ETS/TCF: E-twenty-six transcription factor
GBM: glioblastoma multiforme
GG: ganglioglioma
HGG: high-grade glioma
LGG: low-grade glioma
MAPK: mitogen-activated protein kinase
OIS: oncogene induced senescence
PXA: pleomorphic xanthoastrocyma
Rb: retinoblastoma protein
STR: short tandem repeat
TCGA: The Cancer Genome Atlas
TERT: telomerase reverse transcriptase
