# Supplementary material for: TERT expression is susceptible to BRAF and ETS-factor inhibition in BRAFV600E/TERT promoter double-mutated glioma
Source: Acta Neuropathol Commun. 2019 Aug 7;7:128. doi: 10.1186/s40478-019-0775-6 (PMC6685154; doi:10.1186/s40478-019-0775-6)
Supplement: Supplementary file 1 — Table S1. Antibodies. Table S2. Primers. Table S3. Histopathological, molecular and clinical parameters of the Vienna patient cohort. (DOCX 16 kb) [file 40478_2019_775_MOESM1_ESM.docx]

| **Gabler et al, Table S1: Antibodies** |  |
| --- | --- |
| **Antibody** | **Source** |
| **Western blot** |  |
| **ERK 1/2 (p42/44 MAPK) (#9102)** | Cell signaling, Danvers, MA, USA |
| **P-ERK 1/2 (Thr202/Tyr204) (#9101)** | Cell signaling |
| **MEK 1/2 (D1A5)** | Cell signaling |
| **P-MEK 1/2 (Ser217/221) (#9121)** | Cell signaling |
| **ETS-1 (#6258)** | Cell signaling |
| **P-ETS-1 (Thr38)** | Sigma, Merck, Darmstadt, Germany |
| **β-actin** | Sigma-Aldrich, St.Louis, MO, USA |
| **Rb (4H1)** | Cell signaling |
| **P-Rb (Ser807/811) (#9308)** | Cell signaling |
| **p53 Ab-6 (DO1)** | Labvision/Neomarkers |
| **Cyclin D1 (92G2)** | Cell signaling |
| **p21 (sc-397)** | Santa Cruz, Dallas, TX, USA |
| **ETV1** | Abnova |
| **GABP-α (H-180)** | Santa Cruz |
| **ChIP** |  |
| **IgG, rabbit (control)** | Millipore |
| **H3K27Ac (D5E4-XP)** | Cell signaling |
| **GABP-α (H-180)** | Santa Cruz |
| **ETS1 (D808A)** | Cell signaling |
|  |  |

**Gabler et al, Table S2: Primers**

| **Target gene** | **Sequence** |
| --- | --- |
| **TERT** | forward: 5’-CCAAGTTCCTGCACTGGCTGA-3’  reverse: 5’-TTCCCGATGCTGCCTGAC-3’ |
| **ETS-1** | forward: 5’-GCTGTCTTGTGGATGATG-3’  reverse: 5’-CTTTCTGACCCAGATGAG-3’ |
| **Cyclin D1** | forward: 5’-ACAAACAGATCATCCGCAAACAC-3’  reverse: 5’-TGTTGGGGCTCCTCAGGTTC-3’ |
| **RPL-41** | forward: 5’-CAAGTGGAGGAAGAAGCGA-3’  reverse: 5’-TTACTTGGACCTCTGCCTC-3’ |
| **CDKN2A** | forward: 5’-AGTAACCATGCCCGCATAG-3’  reverse: 5’-TAGGACCTTCGGTGACTGAT-3’ |
| **GABPA** | forward: 5’-CCTGAACTGGTTGCACAGAA-3’  reverse: 5’-ACAAATCATGTCCCCATCG-3’ |
| **GABPB1-L** | forward: 5’-AACCAGTGGAATTGGTCAGC-3’  reverse: 5’-TGTAGGCCTCTGCTTCCTGT-3’ |
| **GABPB1-S** | forward: 5’-AACCAGTGGAATTGGTCAGC-3’  reverse: 5’-ACCGGGTAAAAGACTCCTTAC-3’ |
| **GABPB2** | forward: 5’-AGCAAGTAATGGGGAGTGGA-3’  reverse: 5’-AACCTTACCAGCAGGTACAG-3’ |
| **GFP (ChIP)** | forward: 5’-ACGTAAACGGCCACAAGTTC-3', reverse: 5'-AAGTCGTGCTGCTTCATGTG-3' |
| **TERT promoter (ChIP)** | forward: 5’-CTGCCCCTTCACCTTCCAG-3’  reverse: 5’-CTGCCTGAAACTCGCGCC-3’ |

**Gabler et al, Table S3**

**Histopathological, molecular and clinical parameters of the Vienna patient cohort.** A small patient cohort of eight pediatric patients suffering from different gliomas was analyzed. PXA, pleomorphic xanthoastrocytoma, aPXA anaplastic pleomorphic xanthoastrocytoma; PA, pilocytic astrocytoma; DMG, diffuse midline glioma; wt, wild type; OS, overall survival; DOD, death of disease

| Case No. | Gender | Age (Y) | Histology | WHO Grade | *BRAF* mut | *TERT* mut | OS (months) | Outcome |
| --- | --- | --- | --- | --- | --- | --- | --- | --- |
| 1 | f | 12 | aPXA | III | V600E | C228T | 12 | DOD |
| 2 | f | 12 | PXA | II | V600E | wt | 48 | Alive |
| 3 | f | 14 | aPXA | III | V600E | wt | 23 | DOD |
| 4 | f | 17 | PXA | II | V600E | wt | 31 | Alive |
| 5 | f | 11 | aPXA | III | V600E | wt | 94 | Alive |
| 6 | m | 16 | aPXA | III | V600E | wt | 36 | Alive |
| 7 | m | 4 | PA | I | V600E | wt | 266 | Alive |
| 8 | m | 8 | DMG (H3K27M) | IV | V600E | wt | 19 | Alive |
