# Supplementary material for: TERT expression is susceptible to BRAF and ETS-factor inhibition in BRAFV600E/TERT promoter double-mutated glioma
Source: Acta Neuropathol Commun. 2019 Aug 7;7:128. doi: 10.1186/s40478-019-0775-6 (PMC6685154; doi:10.1186/s40478-019-0775-6)
Supplement: Supplementary file 3 — Figure S1. mRNA expression of ETS transcription factors and downstream targets in tumor tissue. Figure S2. Expression of Ets-factors and TERT in TCGA RNA sequencing data sets. Figure S3. Evaluation of TP53 and CDKN2A/Rb signaling pathways. Figure S4. Activation levels of MEK, ERK and ETS1. Figure S5. Anti-proliferative effects of BRAF inhibitors. Figure S6. Inhibition of TERT expression upon vemurafenib treatment. Figure S7. ETS1 inhibition upon vemurafenib treatment. Figure S8. Changes in expression patterns in patient tissue upon targeted treatment. Figure S9. Ectopic TERT re-expression partly rescues double-mutant glioma cells from YK-4-279- mediated cytotoxicity. Figure S10. Combined BRAF and Ets-factor inhibition. (PDF 786 kb) [file 40478_2019_775_MOESM3_ESM.pdf]

Gabler et al., supplementary figure 1

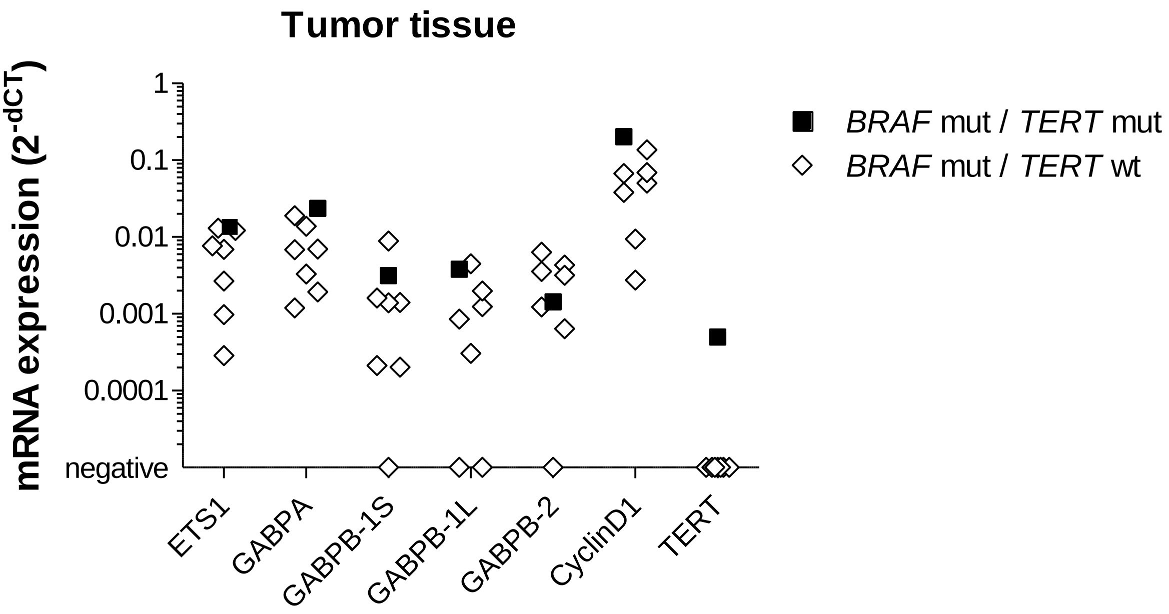

Figure S1. mRNA expression of ETS transcription factors and downstream targets in tumor tissue. mRNA expression of *ETS1*, *GABPA*, *GABPB-1S*, *GABPB-1L and GABPB-2*, as well as of ETS downstream targets *cyclin D1* and *TERT* was assessed using qRT-PCR. Expression was analyzed in tissues of one *BRAF* mut and *TERT* promoter-mut and seven *BRAF* mut/*TERT* promoter-wt glioma patients as indicated. Values are derived from triplicates and given as mean -dCT from *RPL-41* serving as internal control. wt = wild-type, mut = mutant

Gabler et al., supplementary figure 2

A

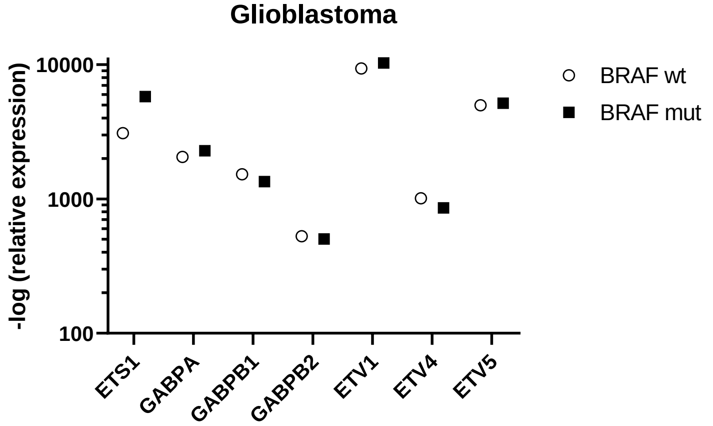

B

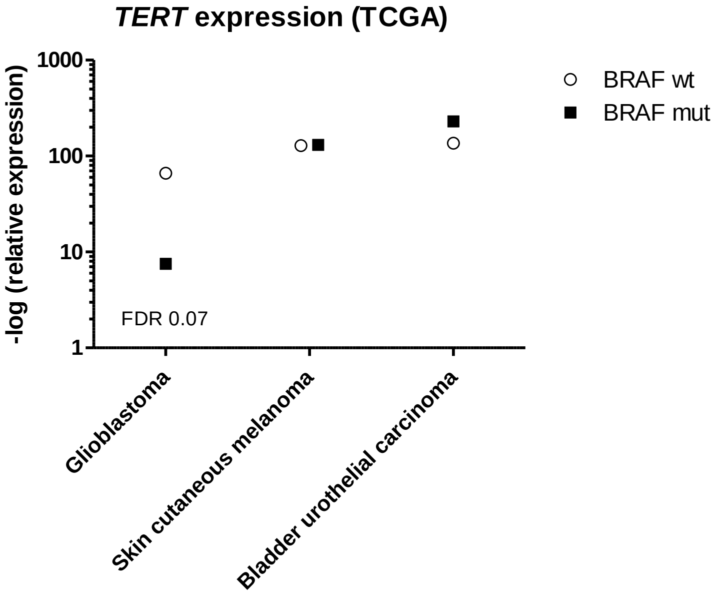

Figure S2. Expression of Ets-factors and *TERT* in TCGA RNA sequencing data sets. Average logarithmic relative Ets-factor **(A)** and *TERT* **(B)** expression in different tumor types stratified for *BRAF* mutation status as calculated by the respective RNA sequencing analysis algorithms (DESeq2). TCGA = The Cancer Genome Atlas, FDR, false discovery rate, wt = wild-type, mut = mutated, Source: TCGA data base

Gabler et al., supplementary figure 3

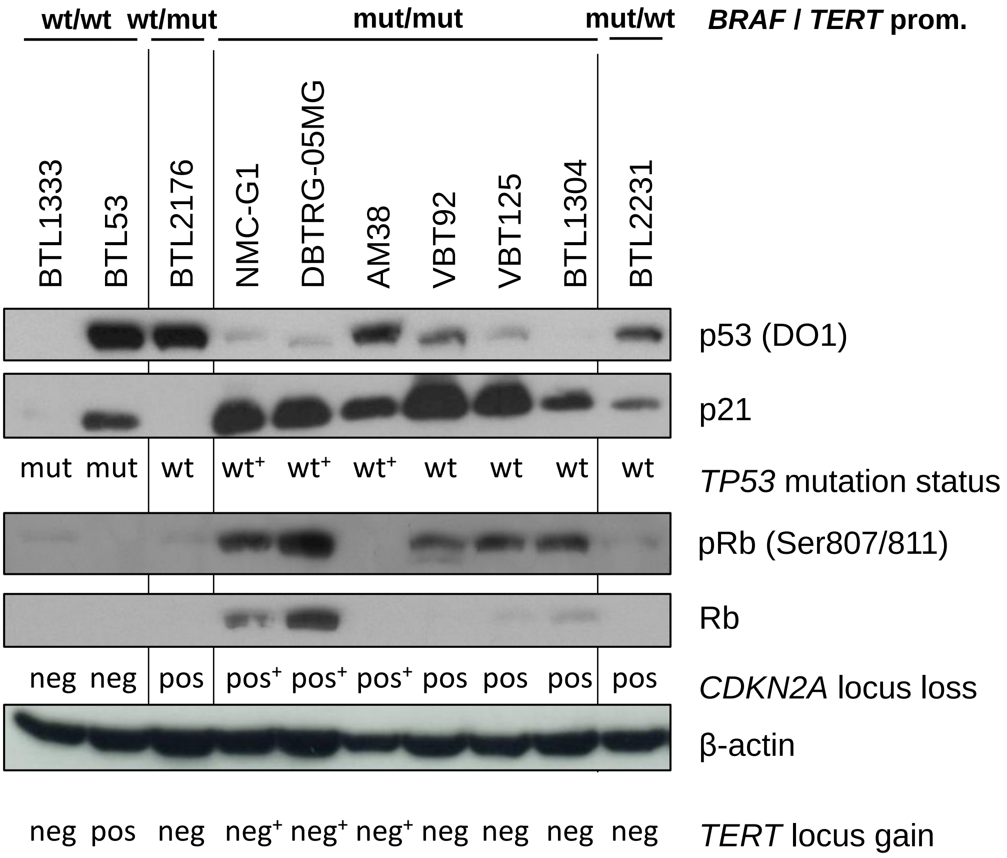

Figure S3. Evaluation of TP53 and CDKN2A/Rb signaling pathways. Protein expression of cell models with the indicated *BRAF* and *TERT* promoter status was analyzed by Western blot analysis. Additionally, *TP53* mutation status (hotspot mutations) and copy number variations of the *CDKN2A* and *TERT* locus are depicted. wt = wild-type, mut = mutated, + Source COSMIC database

Gabler et al., supplementary figure 4

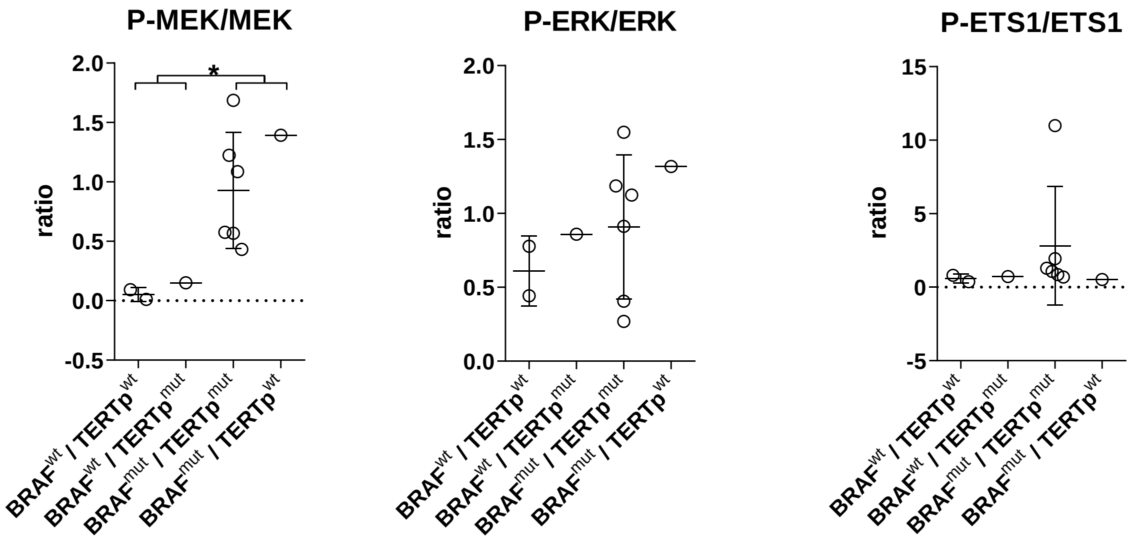

Figure S4. Activation levels of MEK, ERK and ETS1. Data are shown in different *BRAF* and *TERT* promoter genotypes as depicted. Activation changes are calculated from Western blot analysis by dividing the respective phospho-protein level by total protein expression normalized to beta-actin (compare Figure 1B). Values are given as mean +/- SD; Mann-Whitney test (two-tailed): BRAF wt (n=3) vs. BRAF mut (n=7), * p = 0.0167.

Gabler et al., supplementary figure 5

A

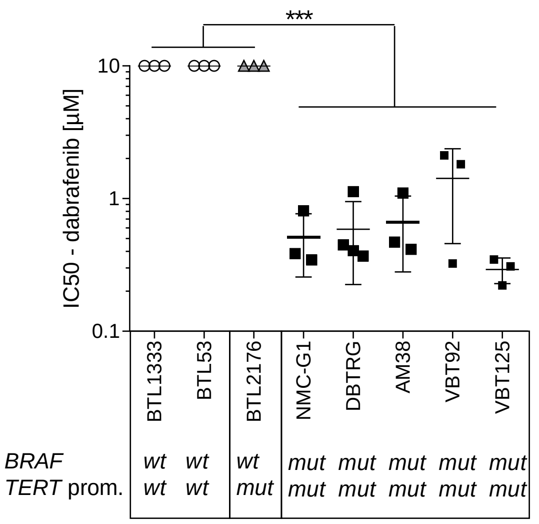

B

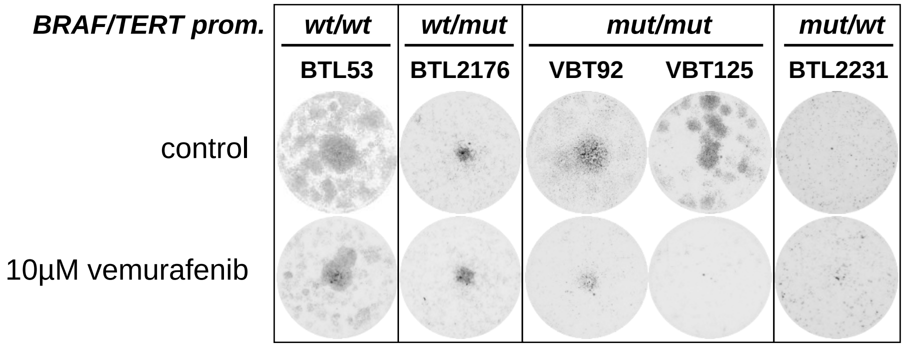

Figure S5. Anti-proliferative effects of BRAF inhibitors. **a)** Cytotoxicity assays were performed treating glioma cells of different *BRAF / TERT* promoter mutation status as depicted with dabrafenib for 72h. Values are given as mean +/- SD from three independent experiments. Ordinary one-way ANOVA (Tukey correction, 0.05 (95% confidence interval)), each *BRAF* wild-type versus each *BRAF*^{V600E} mutated model: *** $p < 0.001$ **b)** Growth inhibition upon vemurafenib treatment. Clone formation assays for one *BRAF* wt/*TERT* promoter wt, one *BRAF* wt/*TERT* promoter mut, two *BRAF* mut/*TERT* promoter mut and one *BRAF* mut/*TERT* promoter wt cell model are depicted. Cells were seeded in duplicates in six-well format at low density and treated with 10µM vemurafenib for seven days. One representative well per condition is shown. wt = wild-type, mut = mutated

Gabler et al., supplementary figure 6

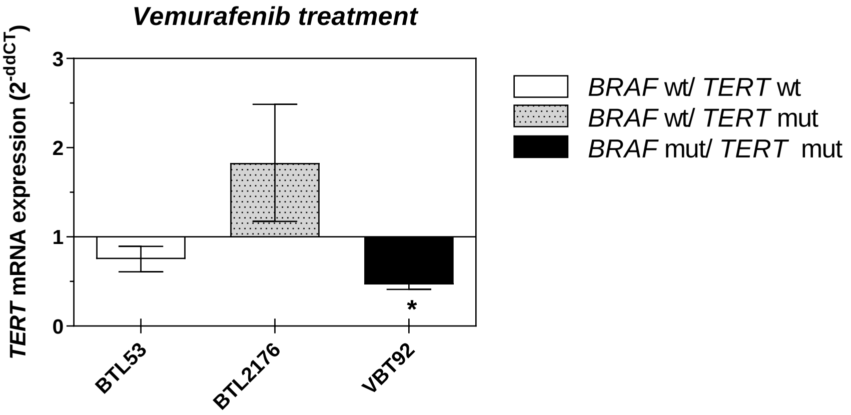

Figure S6. Inhibition of *TERT* expression upon vemurafenib treatment. mRNA expression of *TERT* was analyzed using qRT-PCR. mRNA was isolated from one *BRAF* wt and *TERT* promoter wt, one *BRAF* wt and *TERT* promoter mut and one *BRAF* mut and *TERT* promoter mut cell model as indicated. Cells were treated with 2.5µM vemurafenib for 16h. Results are shown as -ddCT (mean, +/- SD) from *RPL-41* serving as internal control and normalized to the respective untreated control. Unpaired student's t-test of controls vs. treatment wt = wild-type, mut = mutated

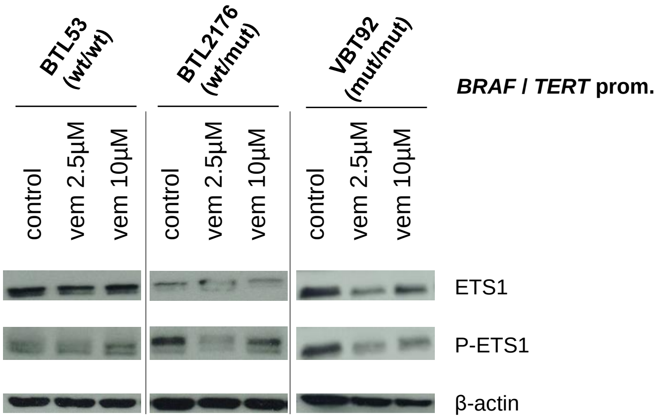

Figure S7. ETS1 inhibition upon vemurafenib treatment. Analysis of altered ETS1 phosphorylation upon 16h vemurafenib treatment by Western blot. β-actin served as internal control. wt = wild-type, mut = mutant, vem = vemurafenib

Gabler et al., supplementary figure 8

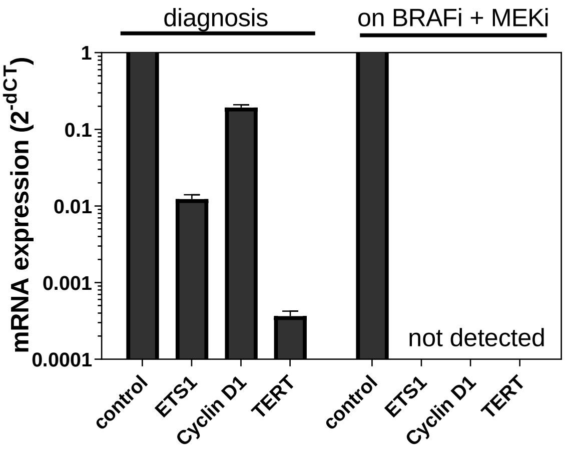

Figure S8. Changes in expression patterns in patient tissue upon targeted treatment. mRNA expression levels of *RPL-41* (control), *ETS1*, *cyclin D1* and *TERT* are shown. mRNA was isolated from the primary tumor (diagnosis) and from tumor tissue derived from a second operation during ongoing BRAF and MEK inhibitor (on BRAFi + MEKi) treatment. Values are given as 2^{-dCT} and normalized to *RPL-41* as internal control (mean +/- SD). BRAFi = BRAF inhibitor, MEKi = MEK inhibitor

Gabler et al., supplementary figure 9

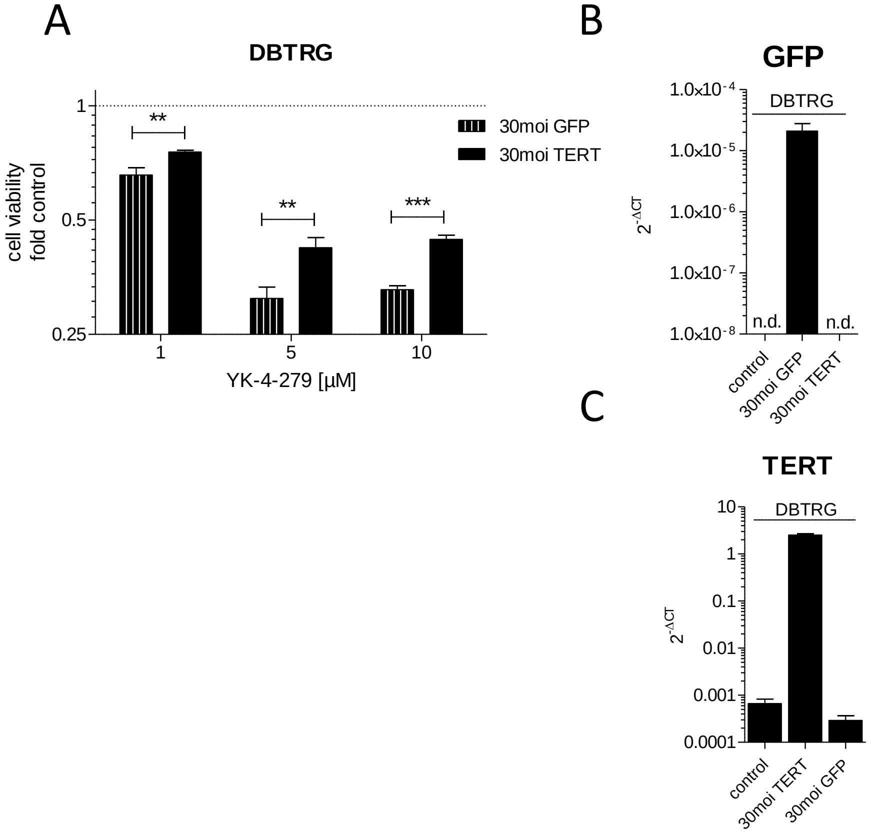

Figure S9. Ectopic *TERT* re-expression partly rescues double-mutant glioma cells from YK-4-279-mediated cytotoxicity. $BRAF^{V600E}$/*TERT* promoter-mutated glioma cells (DBTRG-05-MG) were infected with TERT adenovirus and the GFP adenoviral control at indicated moi. **(A)** Cytotoxicity assay with indicated concentrations of YK-4-279 was performed following virus infection. Values are given as mean +/- SD normalized to the respective untreated control, set to 1. (log2 axis, unpaired student's t-tests: **p<0.01, *** p<0.001) **(B+C)** qRT-PCR from the isolated mRNA was performed and revealed effective virus infection using both adenoviral constructs GFP (B) and TERT (C). Values are given as 2^{-dCT} from RPL-41 serving as internal control. moi = multiplicity of infection, n.d. = not detected, GFP = green fluorescent protein

Gabler et al., supplementary figure 10

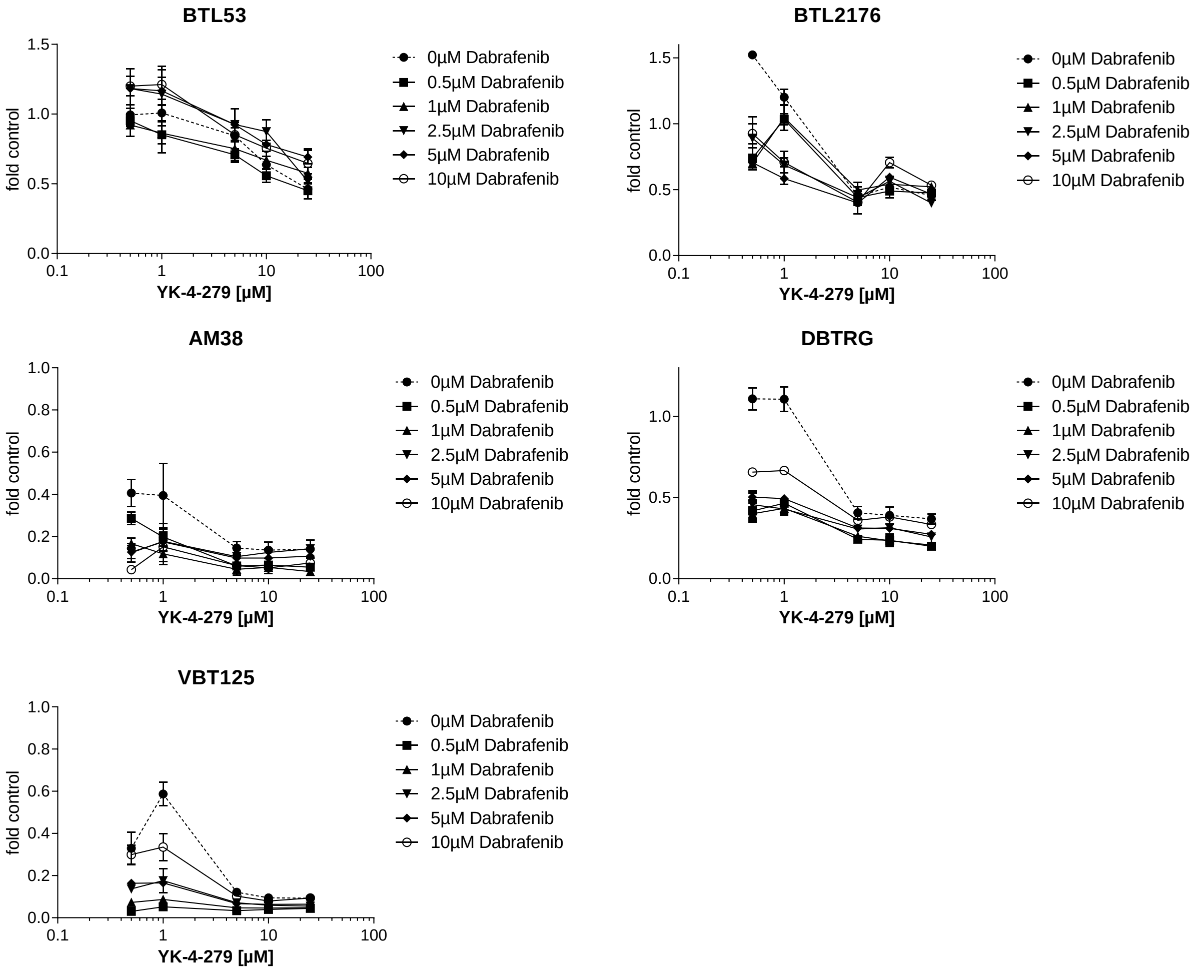

Figure S10. Combined BRAF and Ets-factor inhibition. Cytotoxicity assays were performed in cell models of different genotypes: *BRAF* wild-type / *TERT* promoter wild-type (BTL53), *BRAF* wild-type / *TERT* promoter mutant (BTL2176) and double-mutant (AM38, DBTRG, VBT125). Cell models were treated with combinations of dabrafenib and YK-4-279 for 72h. Values are given as mean +/- SEM from two independent experiments.
